# Frailty Predicts an Increased Risk of End-Stage Renal Disease with Risk Competition by Mortality among 165,461 Diabetic Kidney Disease Patients

**DOI:** 10.14336/AD.2019.0216

**Published:** 2019-12-01

**Authors:** Chia-Ter Chao, Jui Wang, Jenq-Wen Huang, Ding-Cheng Chan, Kuo-Liong Chien

**Affiliations:** ^1^Department of Medicine, National Taiwan University Hospital BeiHu Branch, College of Medicine, National Taiwan University, Taipei, Taiwan; ^2^Geriatric and Community Medicine Research Center, National Taiwan University Hospital BeiHu Branch, Taipei, Taiwan; ^3^Institute of Epidemiology and Preventive Medicine, College of Public Health, National Taiwan University, Taipei, Taiwan; ^4^Nephrology division, Department of Internal Medicine, National Taiwan University Hospital, Taipei, Taiwan; ^5^Department of Medicine, National Taiwan University Hospital ChuTung branch, HsinChu county, Taiwan

**Keywords:** chronic kidney disease, diabetes mellitus, diabetic kidney disease, dialysis, end-stage renal disease, frailty, frail phenotype

## Abstract

To examine the effect of frailty on diabetic kidney disease patients’ risk of progression to end-stage renal disease (ESRD), mortality, and adverse episodes, as whether frailty modifies their risk of developing ESRD and other adverse outcomes remains unclear. We identified 165,461 DKD patients from the Longitudinal Cohort of Diabetes Patients in Taiwan (n=840,000) between 2004 and 2010, classifying them into those without frailty or with 1, 2 and ≥3 frailty components based on a modified version of FRAIL scale. Using Cox proportional hazard regression analysis, we examined the long-term risk of developing ESRD along with their risk of mortality, supplemented by a competing risk analysis against mortality. Among all participants, 66.2% (n=109,586), 27.2% (n=44,986), 5.9% (n=9,799), and 0.7% (n=1090) patients did not have or had 1, 2, and ≥3 frailty components, respectively. After a 4.1-year follow-up, 4.2% patients developed ESRD and 18.5% died. Cox proportional hazard modeling revealed that patients with 1, 2, and ≥3 frailty components had increased risks of developing ESRD (for 1, 2, and ≥3 components, hazard ratio [HR] 1.13, 1.18, and 1.2, respectively) and mortality (HR 1.25, 1.41, and 1.34, respectively), with. 9% and 16% risk elevations for ESRD and mortality per component increase. Competing risk analysis showed that frailty-induced ESRD risk was attenuated partially by mortality in those with moderate frailty. The receipt of palliative care did not attenuate this risk. Frailty increased the risk of ESRD based on a dose-response relationship among DKD patients with risk competition by mortality.

With the rising age of the population and the growing trend of non-communicable disease worldwide, the prevalence of diabetes mellitus (DM) increases progressively over time. Among all diabetic complications, diabetic kidney disease (DKD) is prognostically important, since DKD has been reported to be the most frequent cause of chronic kidney disease (CKD) and end-stage renal disease (ESRD) [1]. DKD is estimated to occur in 20%-40% of DM patients, but the number may vary depending on the changeable combination of the reduced estimated glomerular filtration rate (eGFR), albuminuria, and retinopathy at the time of DM diagnosis [2].

Frailty, a status of increased vulnerability to environmental or endogenous negative factors with impaired physiological reserve, characterizes the degenerative phenotype that occurs during chronological ageing or biologic ageing related to chronic illnesses, especially of those with DM or CKD. Kotsani *et al*. showed that being diabetic is associated with a 1.5-2-fold higher risk of presenting geriatric syndromes at an early time [3]. DM patients are more likely to develop frailty, due to the combination of neuropathy with impaired muscle function, concomitant vascular morbidities with depressed cardiopulmonary reserve, cognitive dysfunction resulting from cerebrovascular events or brain degeneration, and the loss of self-management capacity [4]. In diabetic patients, the presence of frailty correlates with a higher risk of mortality, hospitalization, and healthcare resource consumption, independent of their comorbidities or glycemic control status [5]. This is also the case for CKD/ESRD patients because CKD-related complications, including anemia, mineral bone disorder (MBD), and sarcopenia, could predispose them to develop frailty [6]. Prior studies also affirmed the adverse influences of frailty on CKD patients’ health-related outcomes [7,8].

Since CKD and DM patients are susceptible to the development of frailty, DKD patients are theoretically more inclined to exhibit this degenerative trait. A recent study describes that DKD patients have a significantly higher risk of frailty than non-diabetic CKD patients [9]. However, existing studies rarely focus on the relationship between frailty and the outcomes of DKD patients. Moreover, whether frailty confers detrimental influences on the prognosis of these patients, especially the risk of progression to ESRD, is unclear. Frailty has not been regarded as a risk factor for renal function declining in DKD and CKD patients in the literature [10]. We hypothesized that frailty might be an under-recognized factor associated with renal function decline and impair outcomes in DKD patients. Using a longitudinally assembled diabetic cohort, we aimed to examine the effect of physical frailty on DKD patients’ risk of progression to ESRD, mortality, and adverse episodes including cardiovascular events and healthcare utilization.

## MATERIALS AND METHODS

### Assembly of the cohort with diabetic kidney disease

Adult patients (>20 years) with DM were retrospectively identified from the Longitudinal Cohort of Diabetes Patients between 2004 and 2010. This cohort was established based on a random selection of 120,000 patients with at least a onetime DM diagnosis (International Classification of Disease 9^th^ version - Clinical Modification [ICD-9-CM] code of 250.x) every year during the 7-year study period, with prospective follow-up until the end of study [5]. We further tightened the criteria for diagnosing DM using the presence of at least three times of ICD-9-CM code during out-patient visits or at least one time during in-patient care. Among these patients, we further identified those with CKD, based on the presence of validated codes for at least three times during out-patient visits or at least one time during in-patient care, as described previously [5,11]. Since the current cohort was assembled from a nationwide claim database, Taiwan National Health Insurance database, laboratory data were unavailable for determining eGFR of each participant. However, this database comprehensively documents among covered citizens the demographic profiles, diagnoses, examinations, treatments (including pharmaceuticals, interventions, and operations), and the medical settings in which these management strategies are adopted, and the diagnosis of CKD established using data from this source has been validated against individual data. We defined the day of starting follow-up as the index date, when participants first satisfied the diagnostic criteria of both DM and CKD. Exclusion criteria of this cohort consisted of those with missing demographic data, those receiving any form of renal replacement therapy before the date of fulfilling both diagnoses of DM and CKD, and those with an inadequate length of follow-up (at least 1 year).

Their clinical data, including demographic data, sociologic profile (smoking and alcoholism), comorbidities, treatments with adverse renal influences (any cardiac catheterization and cardiac surgery), and pharmacologic agents with renal and outcome influences including oral anti-diabetic agents (OADs), were documented during the study period. Charlson comorbidity index (CCI) was calculated [12]. The severity of DM was examined using the adjusted diabetic complication severity index (aDCSI) according to previous studies [11]. Proteinuria was defined based on the ICD-9-CM code 791.0. Stage 5 CKD was identified based on the presence of CKD codes and insurance-afforded erythropoietin use among participants. *Per* Taiwan National Health Insurance criterion, erythropoietins are reimbursed only when CKD patients have a serum creatinine higher than 6 mg/dL, equivalent to an eGFR < 15 ml/min/1.73m^2^. The accuracy of this approach has been shown to be excellent [13]. The duration of CKD was defined as the period between the day when participants were first identified as having CKD and the index date. Patients were followed up since the day when they fulfilled both DM and CKD diagnostic criteria, until the development of pre-specified outcomes or the end of follow-up in this study (2011 December 31^st^), whichever occurred first.

### The classification of frailty

We harnessed a construct of the frail phenotype, FRAIL scale but with modifications for ease of identification, in our DKD cohort to determine participants’ frailty status [7,14]. The original FRAIL scale evaluates the status and the severity of frailty using 5 components, Fatigue, Resistance, Ambulation, Illness, and Loss of weight (score range 0-5); the presence of ≥3 components in one individual is designated as being frail, while having more components signifies more severe frailness [15]. For DM or CKD patients, evidence suggests that having FRAIL -recognized frailty is predictive of a higher mortality, hospitalization, and institutionalization compared to those who do not have such traits, independent of other outcome-influencing factors [8,16].

We further operationalization the original FRAIL scale’s 5 components based on a validated clustering of ICD-9-CM codes, with the code combinations available elsewhere [5], since these participants did not undergo interview. In brief, the pre-specified diagnostic codes clustering was selected based on a thorough literature review done previously and several rounds of expert consensus to decrease selection bias. The codes were meant to approximate the items themselves or the underlying pathogenic mechanisms behind these items. For Fatigue, we used the ICD-9-CM codes of malaise and fatigue, neurasthenia, senile asthenia, or general weakness for identification. For Resistance, we used codes of debility or any experiences of fall to screen for those with difficulty in stair climbing. For Ambulation, we used codes of difficulty in walking and gait abnormality to identify those with impairment in ambulation capacity. For Illness, validated codes of different morbidities were used, including hypertension, cancer, chronic obstructive pulmonary disease, acute myocardial infarction, heart failure, angina, asthma, arthritis, stroke, and chronic kidney disease. For Loss of weight, we used codes of malnutrition, feeding difficulty, cachexia, and muscle wasting for identification. The presence of each frailty component was affirmed based on the presence of any of the validated codes in ≥2 out-patient clinics or during hospitalization within the preceding years of before the index date. In this sense, frailty status was ascertained prior to the start of participant follow-up. This approach for identifying frailty was subsequently termed “modified FRAIL scale”. We previously showed that diabetic individuals with frailty based on this modified FRAIL scale examined through this approach exhibited a significantly higher risk of healthcare use and mortality than non-frail ones [5], lending support to its validity.

**Figure 1.**
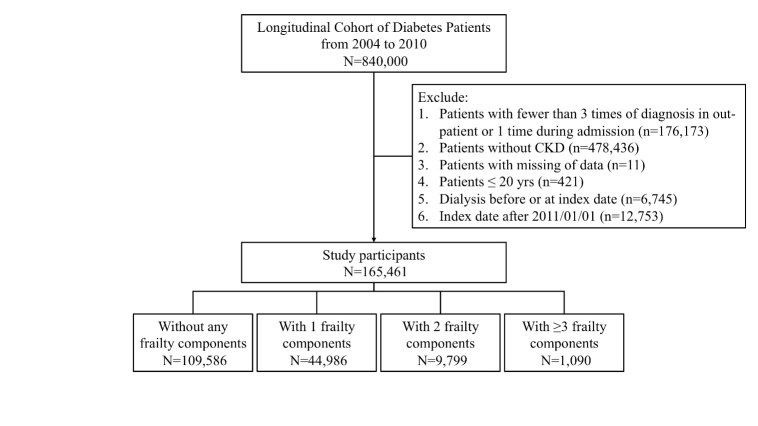
Algorithm of patient selection and analysis in this study. CKD, chronic kidney disease.

**Table 1:** Comparison of participants without and with different severities of frailty at study enrollment.

	Numbers of frailty component				*p-value*
	0 (n=109,586)	1 (n=44,986)	2 (n=9,799)	≥ 3 (n=1090)	
*Demographic profile*					

**Age (years)**	58.1 ± 13.7	67.1 ± 14	73 ± 11.9	77.5 ± 10.9	*< 0.001*
**Sex (Female)**	48,330 (44.1)	20,814 (46.3)	4,756 (48.5)	509 (46.7)	*< 0.001*
**Smoking (%)**	568 (0.5)	282 (0.6)	63 (0.6)	6 (0.6)	*0.04*
**Alcoholism (%)**	1,173 (1.1)	569 (1.3)	124 (1.3)	16 (1.5)	*0.004*

*Duration of CKD (years)*	2.4 ± 3.3	2.6 ± 3.2	2.8 ± 3.3	2.8 ± 3.2	*< 0.001*

< 1	62,106 (56.7)	22,684 (50.4)	4,603 (47)	505 (46.3)	*< 0.001*
1 - 3	11,734 (10.7)	5,746 (12.8)	1,269 (13)	148 (13.6)	
3 - 5	10,849 (9.9)	5,873 (13.1)	1,418 (14.5)	177 (16.2)	
≥ 5	24,897 (22.7)	10,683 (23.8)	2,509 (25.6)	260 (23.9)	

*Comorbidity profile*					

CCI	1.5 ± 1.6	3.1 ± 2.4	4.4 ± 2.5	5.3 ± 2.5	*< 0.001*
aDCSI	0.4 ± 0.8	0.9 ± 1.2	1.2 ± 1.4	1.5 ± 1.5	*< 0.001*
Obesity (%)	1,240 (1.1)	473 (1.1)	86 (0.9)	2 (0.2)	*0.002*
Hypertension (%)	59,617 (54.4)	35,160 (78.2)	8,908 (90.9)	994 (91.2)	*< 0.001*
Hyperlipidemia (%)	36,143 (33)	16,844 (37.4)	3,659 (37.3)	282 (25.9)	*< 0.001*
Chronic liver disease (%)	27,047 (24.7)	14,120 (31.4)	3,452 (35.2)	413 (37.9)	*< 0.001*
COPD (%)	5,126 (4.7)	11,942 (26.6)	4,340 (44.3)	620 (56.9)	*< 0.001*
Atrial fibrillation (%)	7,974 (7.3)	9,457 (21)	3,069 (31.3)	445 (40.8)	*< 0.001*
Acute coronary syndrome (%)	16,009 (14.6)	16,930 (37.6)	5,233 (53.4)	629 (57.7)	*< 0.001*
Cerebrovascular disease (%)	10,260 (9.4)	15,415 (34.3)	5,169 (52.8)	743 (68.2)	*< 0.001*
Peripheral vascular disease (%)	1,919 (1.8)	1,813 (4)	596 (6.1)	82 (7.5)	*< 0.001*
Malignancy (%)	6,438 (5.9)	6,877 (15.3)	2,035 (20.8)	243 (22.3)	*< 0.001*
Parkinsonism (%)	1,206 (1.1)	1,910 (4.3)	839 (8.6)	193 (17.7)	*< 0.001*
Gout (%)	20,837 (19)	12,032 (26.8)	2,895 (29.5)	311 (28.5)	*< 0.001*
Osteoarthritis (any site) (%)	22,031 (20.1)	17,135 (38.1)	5,183 (52.9)	635 (58.3)	*< 0.001*
Osteoporosis (%)	6,447 (5.9)	5,807 (12.9)	1,932 (19.7)	261 (23.9)	*< 0.001*
Proteinuria (%)	1768 (1.6)	738 (1.6)	170 (1.7)	21 (1.9)	*0.684*
*Stage 5 CKD (%)*	187 (0.2)	112 (0.3)	28 (0.2)	2 (0.2)	*< 0.001*

*Treatment with adverse renal influences*					

**Cardiac catheterization (%)**	2,499 (2.3)	3,856 (8.6)	1,099 (11.2)	88 (8.1)	*< 0.001*
**Cardiac surgery (any) (%)**	1,203 (1.1)	2,060 (4.6)	542 (5.5)	39 (3.6)	*< 0.001*

*Medications*					

**Aspirin (%)**	34,212 (31.2)	24,728 (55)	6,870 (70.1)	780 (71.6)	*< 0.001*
β-blockers (%)	52,826 (48.2)	29,747 (66.1)	7,346 (75)	766 (70.3)	*< 0.001*
**ACEi (%)**	36,704 (33.5)	22,420 (49.8)	6,001 (61.2)	619 (56.8)	*< 0.001*
**ARB (%)**	28,749 (26.2)	18,123 (40.3)	4,725 (48.2)	486 (44.6)	*< 0.001*
**Clopidogrel (%)**	2,379 (2.2)	3,920 (8.7)	1,241 (12.7)	118 (10.8)	*< 0.001*
**Statin (%)**	29,006 (26.5)	13,784 (30.6)	3,032 (30.9)	237 (21.7)	*< 0.001*
**Fibrate (%)**	16,093 (14.7)	7,519 (16.7)	1,626 (16.6)	126 (11.6)	*< 0.001*
**NSAID (any) (%)**	105,288 (96.1)	44,411 (98.7)	9,725 (99.2)	1,079 (99)	*< 0.001*
**Allopurinol (%)**	4,880 (4.5)	3,248 (7.2)	849 (8.7)	75 (6.9)	*< 0.001*
**Warfarin (%)**	1,235 (1.1)	1,755 (3.9)	514 (5.3)	56 (5.1)	*< 0.001*
**Benzodiazepine (%)**	66,928 (61.1)	35,376 (78.6)	8,503 (86.8)	968 (88.8)	*< 0.001*
**Anti-depressants (%)**	20,050 (18.3)	14,227 (31.6)	4,198 (42.8)	544 (49.9)	*< 0.001*
**Anti-psychotics (%)**	29,734 (27.1)	18,772 (41.7)	5,492 (56.1)	705 (64.7)	*< 0.001*

*Oral anti-diabetic agents*					

Biguanide (%)	36,531 (33.3)	11,948 (26.6)	2,200 (22.5)	196 (18)	*< 0.001*
Sulfonylurea (%)	36,973 (33.7)	12,232 (27.2)	2,296 (23.4)	193 (17.7)	*< 0.001*
Meglitinide (%)	4,563 (4.2)	1,843 (4.1)	389 (4)	33 (3)	*0.219*
α-glucosidase inhibitor (%)	5,814 (5.3)	2,246 (5)	415 (4.2)	32 (2.9)	*< 0.001*
Thiazolidinedione (%)	3,852 (3.5)	1,202 (2.7)	195 (2)	13 (1.2)	*< 0.001*
DPP4 inhibitors (%)	816 (0.7)	256 (0.6)	60 (0.6)	1 (0.1)	*< 0.001*
Insulin (%)	6,618 (6)	3,073 (6.8)	761 (7.8)	83 (7.6)	*< 0.001*

ACEi, angiotensin-converting enzyme inhibitor; aDCSI, adjusted diabetic complication severity index; ARB, angiotensin receptor blocker; CCI, Charlson comorbidity index; CKD, chronic kidney disease; COPD, chronic obstructive pulmonary disease; DPP4, dipeptidyl peptidase 4; NSAID, non-steroidal anti-inflammatory agent

### Primary and secondary endpoints

The primary outcome of this study was the development of ESRD requiring chronic dialysis, defined as having ICD-9-CM codes of CKD (585.x) and receiving hemodialysis, peritoneal dialysis, or both for ≥3 months consecutively, an approach validated previously. Secondary outcomes included overall mortality, the occurrence of any cardiovascular event [17], hospitalization, and intensive care unit (ICU) admission. For recurrent events, only the first episode was included in the analysis.

### Statistical analysis

We first stratified participants based on the absence or the presence of 1, 2, and ≥3 frailty components of the modified FRAIL scale, and compared the demographic and comorbid profiles, CCI, aDCSI, cardiac procedures, and medication use between participants of different frailty severity. We then examined the distribution of each frailty component among the entire DKD cohort. This was followed by Cox proportional hazard regression analyses (the baseline models), with primary and secondary outcomes as the dependent variables, incorporating demographic profile (age and sex), smoking, alcoholism, comorbidities (obesity, hypertension, hyperlipidemia, chronic liver disease, chronic obstructive pulmonary disease, atrial fibrillation, acute coronary syndrome, cerebrovascular disease, peripheral vascular disease, malignancy, Parkinsonism, gout, osteoarthritis, and osteoporosis), aDCSI, stage 5 CKD or not, cardiac procedures (catheterizations and surgeries), medications they used (aspirin, β-blockers, angiotensin converting enzyme inhibitor, angiotensin receptor blocker, clopidogrel, statin, fibrate, non-steroidal anti-inflammatory agents, allopurinol, warfarin, benzo-diazepine, anti-depressants, anti-psychotics, and anti-diabetic agents), and frailty component counts. Kaplan-Meier technique was applied to draw dialysis-free and other event-free survival curves based on the status and the severity of frailty, with a log -rank test used to compare between groups. A competing risk analysis between ESRD requiring chronic dialysis and mortality as the outcomes was also performed. Finally, sensitivity analyses were conducted to evaluate the contribution of individual frailty component to the pre-specified outcomes.

We further conducted sensitivity analyses, aiming to account for the potential influences of CKD severity and proteinuria. A prior study involving DKD patients showed that the duration of having CKD was closely associated with the severity of CKD and the associated complications [18]. After constructing the baseline models, we further added the variable CKD duration and proteinuria to the regression models for adjustment. Finally, we also conducted a sensitivity analysis, trying to account for the influence of palliative care on the relationship between frailty and the pre-specified outcomes.

**Figure 2.**
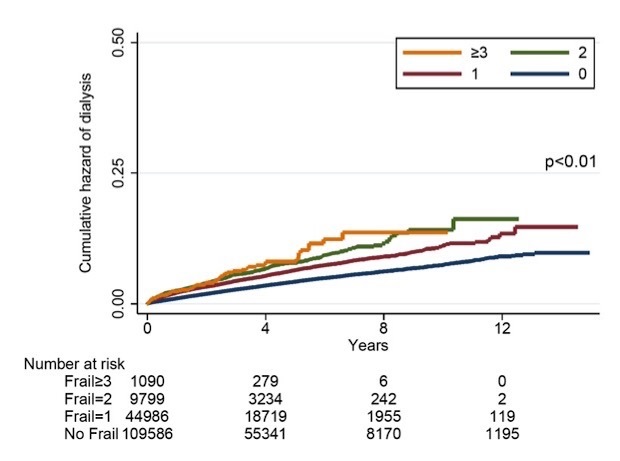
ESRD and chronic dialysis-free survival curves based on frailty status of patients with DKD. DKD, diabetic kidney disease; ESRD, end-stage renal disease

**Table 2:** The distribution of FRAIL component among participants with identified frailty.

	Numbers of frailty component			
	1 (n = 44,986)	2 (n = 9,799)	≥ 3 (n = 1090)
*Components of FRAIL*			

**Fatigue**	17,030 (37.9)	7,801 (79.6)	984 (90.3)
**Resistance**	799 (1.8)	769 (7.9)	363 (33.3)
**Ambulation**	355 (0.8)	559 (5.7)	286 (26.2)
**Illness**	25,697 (57.1)	9,022 (92.1)	1,068 (98)
**Loss of weight**	1,105 (2.5)	1,447 (14.8)	641 (58.8)

### Ethical approval

The protocol of the current study was approved by the institutional review board of National Taiwan University Hospital (NO. 201802063W) and adhered to the Declaration of Helsinki. Informed consent was deemed unnecessary based on the anonymity of the data source by the institutional review board.

**Figure 3.**
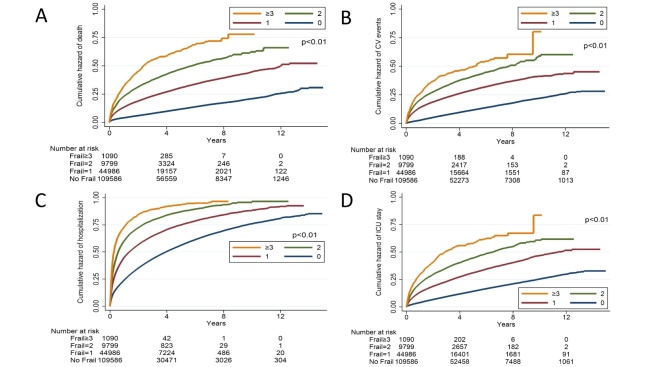
Kaplan-Meier analyses based on the secondary endpoints. Overall (A), cardiovascular event-free (B), hospitalization-free (C), and ICU-free (D) survival curves based on frailty status of patients with DKD. CV, cardiovascular; DKD, diabetic kidney disease; ICU, intensive care unit

## RESULTS

From the Longitudinal Cohort of Diabetic Patients, we screened 840,000 diabetic patients during the study period; after tightening the diagnostic criteria for DM and excluding pediatric cases, those with missing data, without CKD or with pre-existing ESRD, a total of 165,461 DKD patients were enrolled and entered into the analysis (Fig. 1). Among these patients, 109,586 (66.2%) did not present frailty using the modified FRAIL scale, while 44,986 (27.2%), 9,799 (5.9%), and 1,090 (0.7%) had 1, 2, and ≥3 frailty components. DKD patients with frailty were of higher age, more likely to be male, and had a significantly higher prevalence of comorbidities than those without. Similarly, DKD patients with more severe frailty were of higher age, more likely to be male, had more comorbidities, and more likely to receive most medications as examined (Table 1). Those with greater frailty severity (2 or ≥3 components) had a significantly longer duration of CKD compared to those without or with milder frailty (for those without, with 1, 2, and ≥3 components, 2.4 ± 3.3 vs. 2.6 ± 3.2 vs. 2.8 ± 3.3 vs. 2.8 ± 3.2 years, *p* < 0.001) (Table 1). The prevalence of proteinuria did not differ according to the number of frailty components (for those without, with 1, 2 and ≥3 components, 1.6% vs. 1.6% vs. 1.7% vs. 1.9%, *p* = 0.68).

For those with only 1 frailty component, Illness was the most common domain present (57.1%), followed by Fatigue (37.9%) (Table 2). More than 90% DKD patients with ≥2 frailty component scored positive in the Illness domain, while Ambulation factor was the least prevalent (Table 2).

**Table 3:** Cox proportional hazard regression with primary and secondary outcomes.

Outcomes	Event	PY	ID*	Crude		Model 1%		Model 2#		Model 3&		Competing risk	
				HR	95% CI	HR	95% CI	HR	95% CI	HR	95% CI	HR	95% CI
***Entering chronic dialysis***													

*Frailty component count*													

0	4,107	479,967.2	8.6	1	-	1	-	1	-	1	-	1	-
1	2,261	165,463.9	13.7	1.57	1.49-1.65^a^	1.13	1.06-1.2^b^	1.14	1.07-1.22^a^	1.14	1.07-1.22^a^	1.09	1.03-1.16^b^
2	535	29,994.1	17.8	2	1.82-2.19^a^	1.18	1.06-1.3^b^	1.2	1.09-1.34^b^	1.2	1.08-1.33^b^	1.07	0.96-1.19
≥ 3	56	2,686.8	20.8	2.27	1.74-2.95^a^	1.2	0.91-1.57	1.22	0.93-1.6	1.2	0.91-1.57	0.97	0.72-1.31
Every 1 component				1.44	1.4-1.49^a^	1.09	1.05-1.14^a^	1.1	1.06-1.15^a^	1.1	1.05-1.15^a^	1.04	0.99-1.09

***Mortality***													
*FRAIL component count*													
0	12,583	487,396.9	25.8	1	-	1	-	1	-	1	-		
1	13,032	168,415.5	77.4	2.9	2.83-2.97^a^	1.25	1.21-1.29^a^	1.26	1.22-1.3^a^	1.26	1.22-1.3^a^		
2	4,330	30,562.4	141.7	5.09	4.92-5.27^a^	1.41	1.35-1.47^a^	1.42	1.36-1.48^a^	1.42	1.36-1.48^a^		
≥ 3	661	2,729	242.2	8.19	4.57-8.86^a^	1.34	1.23-1.46^a^	1.35	1.24-1.47^a^	1.35	1.24-1.47^a^		
Every 1 component				2.24	2.21-2.27^a^	1.16	1.14-1.18^a^	1.16	1.14-1.19^a^	1.16	1.14-1.19^a^		

***Cardiovascular events***													

*FRAIL component count*													

0	11,582	459,723	25.2	1	-	1	-	1	-	1	-		
1	10,974	144,045.6	76.2	2.9	2.83-2.98^a^	1.4	1.35-1.44^a^	1.41	1.36-1.45^a^	1.41	1.36-1.45^a^		
2	3,074	24,222.2	126.9	4.61	4.43-4.8^a^	1.48	1.41-1.55^a^	1.49	1.43-1.57^a^	1.49	1.43-1.57^a^		
≥ 3	386	2,050.8	188.2	6.42	5.8-7.1^a^	1.55	1.39-1.7^a^	1.57	1.41-1.74^a^	1.56	1.41-1.74^a^		
Every 1 component				2.18	2.15-2.21^a^	1.22	1.2-1.25^a^	1.23	1.2-1.25^a^	1.23	1.2-1.25^a^		

***Hospitalization***													

*FRAIL component count*													

0	56,620	310,452.3	182.4	1	-	1	-	1	-	1	-		
1	30,478	83,907.9	363.2	1.81	1.78-1.83^a^	1.17	1.15-1.19^a^	1.18	1.16-1.19^a^	1.18	1.16-1.19^a^		
2	7,556	11,834.1	638.5	2.8	2.73-2.87^a^	1.28	1.24-1.31^a^	1.29	1.25-1.32^a^	1.29	1.25-1.32^a^		
≥ 3	905	843.8	1072.6	4.04	3.78-4.32^a^	1.36	1.27-1.46^a^	1.38	1.28-1.47^a^	1.38	1.28-1.47^a^		
Every 1 component				1.7	1.68-1.71^a^	1.14	1.12-1.15^a^	1.14	1.13-1.15^a^	1.14	1.13-1.15^a^		

***ICU admission***													

*FRAIL component count*													

0	14,369	460,577.7	31.2	1	-	1	-	1	-	1	-		
1	11,970	149,386.7	80.1	2.48	2.42-2.54^a^	1.26	1.22-1.3^a^	1.27	1.23-1.31^a^	1.27	1.23-1.31^a^		
2	3,513	25,638.9	137	4.07	3.93-4.23^a^	1.37	1.31-1.43^a^	1.38	1.33-1.45^a^	1.38	1.33-1.45^a^		
≥ 3	483	2,163.1	223.3	6.26	5.72-6.86^a^	1.37	1.25-1.51^a^	1.39	1.26-1.53^a^	1.39	1.26-1.53^a^		
Every 1 component				2.05	2.02-2.08^a^	1.17	1.14-1.19^a^	1.17	1.15-1.19^a^	1.17	1.15-1.19^a^		

CI, confidence interval; HR, hazard ratio; ICU, intensive care unit; ID, incidence density; PY, person-year

* per 1000 patient-year

% Baseline model

# Baseline model, adding duration of CKD

& Baseline model, adding duration of CKD and proteinuria

a p < 0.001

b p < 0.01

**Table 4:** Cox proportional hazard regression with receiving palliative care as the dependent variable.

Outcomes	Event	P-Y	ID*	Crude		Model 1%		Model 2#		Model 3&	
				HR	95% CI	HR	95% CI	HR	95% CI	HR	95% CI
***Receiving palliative care***													

*FRAIL component count*													

0	556	487,309.9	1.14	1	-	1	-	1	-	1	-
1	405	168,349.2	2.41	2.08	1.83-2.36^a^	0.99	0.85-1.15	1	0.85-1.16	1	0.85-1.16
2	119	30,535.7	3.9	3.29	2.7-4.01^a^	1.12	0.89-1.42	1.14	0.9-1.43	1.14	0.9-1.43
≥ 3	8	2,727.3	2.93	2.38	1.18-4.78^c^	0.6	0.29-1.23	0.6	0.29-1.23	0.6	0.29-1.23
Every 1 component				1.78	1.64-1.92^a^	1	0.9-1.11	1.01	0.91-1.12	1.01	0.91-1.12

CI, confidence interval; HR, hazard ratio; ICU, intensive care unit; ID, incidence density; P-Y, person-year

* per 1000 patient-year

% Baseline model

# Baseline model, adding duration of CKD

& Baseline model, adding duration of CKD and proteinuria

a p < 0.001

b p < 0.01

c p < 0.05

After a mean 4.1 years of follow-up, 6,959 and 30,606 patients developed ESRD requiring chronic dialysis and mortality, with an incidence of ESRD and mortality approximately 4.2% and 18.5%, respectively (Table 3). In addition, 26,061 (15.7%) participants developed cardiovascular events, while 95,559 (57.8%) and 30,335 (18.2%) had experienced hospitalization and ICU admission, respectively. Kaplan-Meier analysis showed that DKD patients with 1, 2, and ≥3 frailty components had a progressively higher incidence of ESRD during follow-up compared to non-frail ones (*p*<0.01) (Fig. 2). DKD patients with 1, 2, and ≥3 frailty components similarly had a higher incidence of mortality (*p*<0.01), cardiovascular events (*p*<0.01), hospitalization (*p*<0.01), and ICU admission (*p*<0.01), in a dose-responsive manner (*p*<0.01) (Fig. 3). Cox proportional hazard regression, incorporating frailty in a categorical fashion, revealed that having 1 (hazard ratio (HR) 1.13, 95% confidence interval (CI) 1.06-1.2) and 2 (HR 1.18, 95% CI 1.06-1.3) frailty components was associated with a higher risk of ESRD, while having ≥3 frailty components exhibited a trend toward a higher risk (HR 1.2, 95% CI 0.91-1.57), compared to those without, independent of other confounding variables (Table 3). For secondary outcomes, we found that having 1, 2, and ≥3 frailty components were associated with a significantly higher risk of mortality, cardiovascular events, hospitalization, and ICU admission, compared to those without, in a dose-dependent manner (Table 3). If we treated frailty in a continuous fashion, for every frailty component increase, there was a 9% increase in the risk for ESRD, and 16%, 22%, 14%, and 17% increase in the risk for mortality, cardiovascular events, hospitalization, and ICU admission.

A competing risk analysis between the endpoint of ESRD and mortality, incorporating frailty as a categorical variable, showed that the association between frailty and the risk of ESRD requiring chronic dialysis disappeared in those with 2 and ≥3 frailty components, if mortality was considered, but persisted only in those with mild frailty (1 component) (Table 3). On the other hand, the risk of cardiovascular events, hospitalization, and ICU admission associated with frailty were not attenuated by further considering mortality.

Sensitivity analyses incorporating the duration of CKD and proteinuria, with frailty in a categorical fashion, showed that DKD patients with 1 and 2 frailty components still had a significantly higher risk of entering ESRD than those without frailty, while those with ≥3 components did not (for those with 1, 2, and ≥3 components, HR 1.14, 1.2, and 1.22, *p* < 0.001, < 0.01, and > 0.05, respectively) (Table 3). Finally, we conducted sensitivity analyses, using the receipt of palliative care as an additional outcome in these participants. We discovered that having different severities of frailty was not associated with an increased probability of receiving palliative care among DKD patients (for 1, 2, and ≥3 frailty components, HR for receiving palliative care 0.99, 1.12, and 0.6, respectively; all p values > 0.05) (Table 4).

## DISCUSSION

**Figure 4.**
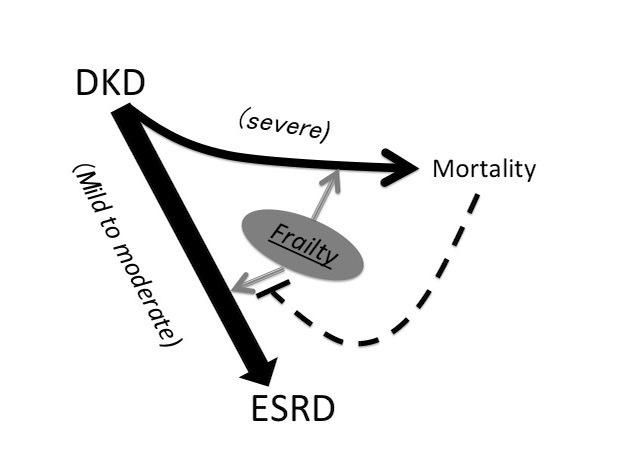
An illustrative diagram showing the inter-relationship between different severities of frailty and the risk of ESRD and mortality based on findings of this study. DKD, diabetic kidney disease; ESRD, end-stage renal disease

In the current study, we identified 0.7% and 32% of 165,461 DKD patients that had full-fledged and milder frailty according to the modified FRAIL scale, respectively. Those with higher frailty severity had significantly higher age, more comorbidities, and were more likely to receive multiple medications than those without or milder frailty. We found that frailty occurring near the diagnosis of DKD predicted a significantly higher risk of progressing to ESRD, except those with the most severe frailty, while the risk increased with higher frailty severity. Furthermore, the associated risk was neutralized by mortality when DKD patients had severe frailty. These findings were not influenced by the probability of receiving palliative care. On the other hand, DKD patients across all frailty severity had a significantly higher risk of mortality, cardiovascular events, hospitalization, and ICU admission compared to those without frailty. Our findings depict frailty as an important risk factor for renal progression and may shed some light on the complex conundrum of renal care in these patients (Fig. 4).

In this study, we used administrative health data for frailty mobilization, an approach that can be advantageous. Frailty screening is uncommon during clinical practice, and data regarding the epidemiology and prognostic significance of frailty mostly come from cohort studies of various sizes. Using administrative data to identify frailty can facilitate a more comprehensive and population-based understanding of the significance of frailty. Frailty studies using administrative data frequently aimed to construct multi-variable frailty indices, using variables (or deficits) from all domains with summation and identifying the threshold for frailty based on the population distribution of deficit accumulations. However, researchers increasingly used diagnostic codes from administrative database for operationalization of frail phenotype and/or its components, and our approach was similar to theirs. Soong and colleagues estimated the prevalence of frailty components, including falls, fractures, immobility, and psychiatric disorders, based on ICD-10 diagnostic code clusters from a secondary care administrative data [19]. Another group also harnessed clusters of diagnoses from administrative data to create frailty defining criteria, termed Johns Hopkins Adjusted Clinical Groups frailty indicator; this indicator has been shown to predict the risk of hospitalization and mortality among different populations [20]. Similarly, McAlsiter *et al*. derived and validated a Hospital Frailty risk Score using ICD-10 diagnostic codes from a large electronic health record database in England, based on different combinations of diagnostic clusters to approximate frailty syndromes [21]. They showed that results from the ICD code-based frailty were correlated well with the degree of functional impairment, and higher scores predicted an increased risk of mortality. In this study, we also used ICD codes-based diagnoses combination to identify those with frailty, using the construct of FRAIL scale for mobilization. Previously we have validated our code combination in a large group of diabetic patients, by showing that participants with frailty according to this “modified FRAIL scale” exhibited a higher risk of mortality, hospitalization, and healthcare utilization than those without [5]. Consequently, we believe that our approach for frailty identification may be credible and applicable for use in administrative healthcare dataset.

Prior studies addressing the role of frailty in CKD patients mainly involve the increasing incidence of frailty and the associated pathobiology, the awareness and assessment methodology of frailty in this population, and adverse consequences brought by the presence or progression of frailty [22]. More recently, the construct of frailty has been used to facilitate the decision of initiating renal replacement therapy or not [23,24]. Researchers have proposed several reasons potentially responsible for earlier initiation of dialysis in frail patients; components of frailty, including malnutrition related weight loss and functional decline, might prompt nephrologists to consider dialysis earlier than expected for relieving these symptoms which will otherwise be attributed to uremic status [25]. Alternatively, low muscle origin creatinine generation and hypocreatininemia can accompany sarcopenia, a frequent companion of frailty, leading to difficulty in estimating true GFR level [26,27]. The relationship between early dialysis initiation and poorer survival among CKD patients may be confounded at least partially by the fact that frail CKD patients are dialyzed earlier than non-frail ones [28]. However, this speculation fails to consider the possibility that frail CKD patients are also at a higher risk of mortality than non-frail ones, therefore altering their risk of subsequent dialysis initiation. To resolve this, the relationship between frailty and dialysis initiation needs to be evaluated in the context of a competing probability between ESRD and mortality. In this study, we discovered that having frailty was predictive of a significantly higher risk of developing ESRD, except in its most severe form, but the risk was attenuated partially by the event of mortality (Table 3). Our findings underline the possibility that CKD patients may be more susceptible to developing ESRD if they are mildly frail, while more susceptible to mortality if they have severe frailty, compared to non-frail ones (Figure 4). This presumption can assist in disentangling the heterogeneous population of frail CKD patients by dividing them into those who are ESRD-prone and others who are mortality-prone.

Whether frailty participates in worsening the renal function or is simply a marker for those with more severe renal impairment, is still under active debate. Its classification criteria, such as weight loss and ambulation difficulty, may more likely be satisfied when CKD patients develop hypoalbuminemia, anemia, and other cardiovascular morbidities, including cardiac dysfunction and peripheral vascular disease [7,29]. Renal osteodystrophy and uremic sarcopenia impair musculoskeletal health, bone integrity, and patients’ exercise capacity [14,30]. These abnormalities favor the perspective that frailty acts as a marker for more severe renal complications. On the other hand, the presence of frailty, uremic sarcopenia, and a falsely low serum creatinine may delay the clinical recognition of on-going renal deterioration, thereby creating a relatively longer timeline during which nephrotoxin exposure can occur, than those without frailty. Moreover, frailty has been reported to be correlated with a higher degree of inflammation and oxidative stress among CKD patients, constituting an interconnected vicious cycle that propagates and persists even after ESRD status occurs [31,32]. In this sense, we believe that the two theories between frailty and renal function worsening described above are both reasonable.

Now that frailty can be an important factor modifying the renal outcome of DKD patients, presumably, interventions aiming to improve frailty can be harnessed to decelerate the progression of renal failure. Increased energy and amino acid supplementation have been found to restore muscle mass and improve frailty among undernourished frail individuals, while dedicated exercise programs can also be beneficial for frail subjects [33,34]. However, high protein intake is a potential risk factor for renal worsening among CKD patients, and increased energy intake also predisposes diabetic patients to glycemic dysregulation. Consequently, an optimized nutritional program that balances the protein and energy needed for both CKD and frailty management is urgently required for these frail DKD patients, as they are the ones that tend to have the worst outcome compared to those without frailty.

This study has its strengths and limitations that should not be neglected. The size of the DKD cohort, the validated strategy, and the comprehensiveness of confounding variable registration prominently add to the credibility of our findings. However, the relatively low prevalence of frailty in our cohort suggests that DKD patients we enrolled had better physical performance compared to others in the literature, and the severity of CKD could be less severe. The severity of CKD in this study was estimated based on diagnostic codes instead of laboratory data, which could lead to variations in CKD staging, although we have tried to account for this issue through additional adjustment for proteinuria and the duration of CKD in the sensitivity analyses. Variables with potential influences on renal outcomes, such as dietary constituents, herbal medicine consumption, and blood pressure control, were not recorded in this study, and therefore, their effect could not be completely excluded. Our participants were all Taiwanese; thus, ethnicity homogeneity could also limit the applicability of our findings. Nonetheless, our study could be among the few ones that examined frailty as a renal prognosis factor of DKD patients against the odds of mortality, and our results could undoubtedly shed light on the importance of frailty in this population.

### Conclusion

Among a cohort of 165,461 DKD patients, we found that 32.7% of them had mild to severe frailty based on a modified FRAIL scale. After adjusting for a multitude of renal outcome-modifying factors, we revealed that frailty was associated with an increased risk of developing ESRD, requiring chronic dialysis compared to non-frail ones, after 4.1 years of follow-up. The risk was attenuated partially by mortality when these patients had a moderate degree of frailty. Frailty also increased the risk of mortality, hospitalization, cardiovascular events, and ICU admission among DKD patients. Further studies are needed to confirm our findings.
